# Using a Seed-Network to Query Multiple Large-Scale Gene Expression Datasets from the Developing Retina in Order to Identify and Prioritize Experimental Targets

**DOI:** 10.4137/bbi.s417

**Published:** 2008-02-01

**Authors:** Laura A. Hecker, Timothy C. Alcon, Vasant G. Honavar, M. Heather West Greenlee

**Affiliations:** 1 Interdepartmental Neuroscience Program, Iowa State University, Ames, IA 50011; 2 Bioinformatics and Computational Biology Graduate Program, Iowa State University, Ames, IA 50011; 3 Department of Computer Science, Bioinformatics and Computational Biology Graduate Program, Center for Computational Intelligence, Learning and Discovery, Iowa State University, Ames, IA 50011; 4 Department of Biomedical Sciences, Interdepartmental Neuroscience Program, Bioinformatics and Computational Biology Graduate Program, Center for Computational Intelligence, Learning and Discovery, Iowa State University, Ames, IA 50011

**Keywords:** gene expression, gene network, cell fate determination, retina, photoreceptor

## Abstract

Understanding the gene networks that orchestrate the differentiation of retinal progenitors into photoreceptors in the developing retina is important not only due to its therapeutic applications in treating retinal degeneration but also because the developing retina provides an excellent model for studying CNS development. Although several studies have profiled changes in gene expression during normal retinal development, these studies offer at best only a starting point for functional studies focused on a smaller subset of genes. The large number of genes profiled at comparatively few time points makes it extremely difficult to reliably infer gene networks from a gene expression dataset. We describe a novel approach to identify and prioritize from multiple gene expression datasets, a small subset of the genes that are likely to be good candidates for further experimental investigation. We report progress on addressing this problem using a novel approach to querying multiple large-scale expression datasets using a ‘seed network’ consisting of a small set of genes that are implicated by published studies in rod photoreceptor differentiation. We use the seed network to identify and sort a list of genes whose expression levels are highly correlated with those of multiple seed network genes in at least two of the five gene expression datasets. The fact that several of the genes in this list have been demonstrated, through experimental studies reported in the literature, to be important in rod photoreceptor function provides support for the utility of this approach in prioritizing experimental targets for further experimental investigation. Based on Gene Ontology and KEGG pathway annotations for the list of genes obtained in the context of other information available in the literature, we identified seven genes or groups of genes for possible inclusion in the gene network involved in differentiation of retinal progenitor cells into rod photoreceptors. Our approach to querying multiple gene expression datasets using a seed network constructed from known interactions between specific genes of interest provides a promising strategy for focusing hypothesis-driven experiments using large-scale ‘omics’ data.

## Introduction

Blinding degenerative retinal diseases including retinitis pigmentosa and macular degeneration are characterized by a loss of photoreceptors. At present there is no way to replace retinal cells lost due to disease or injury because differentiated retinal cells are unable to regenerate. Various stem and/or progenitor cell populations have been proposed as a potential source of transplantable cells to replace lost cells in the damaged retina. The retina is composed of five major neuronal types and one glial cell type that all originate from the same pool of progenitor cells. The rod photoreceptors, the most numerous among retinal cells, together with cone photoreceptors, are responsible for transduction of light and are required for vision. Recent studies demonstrate that post-mitotic rod precursors are able to differentiate and fully integrate into the damaged retina, whereas less differentiated cells are not (Maclaren et al. 2006). Understanding the network of genes that orchestrate the differentiation of retinal progenitors may make it possible to bias expanded stem cell populations to generate rod precursors.

Large-scale gene expression profiling is aimed at helping to understand how genes influence each other in networks, which then control cell fate commitment and differentiation. There are a number of published studies that have profiled changes in gene expression during normal retinal development (Blackshaw et al. 2001; Blackshaw et al. 2004; Diaz et al. 2003; Dorrell et al. 2004; Yu et al. 2003). However, the large number of genes profiled at comparatively few time points or conditions presents significant statistical challenges in inference of genetic networks from any given dataset. One way to more effectively understand relationships between genes is to increase the number of expression measurements for a given gene, and/or focus the investigation on a small number of genes of interest (or between clusters of genes that have similar expression profiles) (Zhou and Mao, 2006). Approaches that leverage existing biological knowledge (e.g. experimentally determined interactions among a small set of genes) to focus the analysis of data from large-scale gene expression studies are beginning to be explored (Bader, 2003; Cabusora et al. 2005; Can et al. 2005; Dougherty et al. 2000; Hashimoto et al. 2004; Shmulevich et al. 2002). Of particular interest is the use of such approaches to prioritize targets for further investigation using traditional experimental techniques.

In this study, we explore an approach to integrated analysis of multiple gene expression datasets in the context of a set of experimentally established relationships between genes. We used the data from five previously published expression studies (Akimoto et al. 2006; Blackshaw et al. 2004; Dorrell et al. 2004; Liu et al. 2006; Zhang et al. 2006) that have provided gene expression data for large numbers of genes under comparable conditions. We queried the resulting datasets using a ‘seed network’ of genes known to play key roles during rod genesis and differentiation (Ahmad et al. 1998; Chen et al. 1997; Cheng et al. 2004; Furukawa et al. 2002; Furukawa et al. 1997; Green et al. 2003; Mears et al. 2001; Nishida et al. 2003; Pennesi et al. 2003; Rutherford et al. 2004; Zhang et al. 2004). We hypothesize that additional genes important for rod genesis and differentiation are likely to be highly positively or negatively correlated with genes that belong to the seed network. We generated a list of such candidate genes based on the correlation of their expression with genes in the seed network. To increase the robustness of analysis, we selected those genes that are correlated with multiple seed network genes in at least two of the five datasets. We further prioritized the resulting candidate genes, based on their gene ontology annotations, evidence of their membership in known cellular signaling pathways, and biological knowledge (whenever such knowledge is available). Using this approach, we identified genes whose expression levels are correlated with multiple genes of interest. Of these, 986 genes are positively correlated with multiple genes of interest and 531 are negatively correlated with multiple genes of interest. We short-listed 7 genes or groups of genes from the list of 986 candidates for inclusion in a hypothesized rod network that extends our seed network. We believe that our results demonstrate the utility of querying multiple large-scale gene expression profiles using a seed network to prioritize genes for further investigation using detailed experimental studies.

## Materials and Methods

### Datasets measuring gene or protein expression in the developing mouse retina

Datasets measuring gene or protein expression in the developing mouse retina at multiple time points include: SAGE (serial analysis of gene expression) of whole retina (Blackshaw et al. 2004), two Affymetrix microarrays of whole retina using the Mu74Av2 chip (hereafter referred to as Mu74Av2_1 (Dorrell et al. 2004) and Mu74Av2_2 (Liu et al. 2006), one cDNA microarray of whole retina (Zhang et al. 2006), one Affymetrix microarray of only developing rod progenitors using the MOE430.2.0 chip (Akimoto et al. 2006), and 2D PAGE (polyacrylamide gel electrophoresis) of whole retina (Barnhill and Greenlee personal communication).

### ID mapping

Genes or proteins from each of these datasets were matched by Entrez gene ID. These IDs were determined using NCBI’s gene database (http://www.ncbi.nlm.nih.gov/entrez/query.fcgi?CMD=search&DB=gene) (Maglott et al. 2007) and WebGestalt (http://bioinfo.vanderbilt.edu/webgestalt/) (Zhang et al. 2005). One difficulty with cross-platform studies is that each microarray probe or SAGE tag must be mapped to some common set of gene identifiers. It is very often the case that more than one probe or tag will be mapped to the same gene, with the possibility that the different probes or tags represent alternative splicings of the same gene. There are three possible approaches to this problem. One is to keep expression measurements for each probe or tag separate, as different ‘versions’ of a gene. This fails to solve the problem since there is currently no good way to match equivalent splicings of the same gene across platforms. Another approach is to get rid of any genes with ambiguous mappings. This approach ends up throwing away a lot of potentially helpful data. The third possibility is to combine the expression measurements for probes or tags that map to the same gene. The drawback of this method is that if the different probes or tags represent valid alternative splicings of the same gene, then these different splicings may in fact have different biological roles and hence different patterns of expression. However it at least provides an approximate matching and avoids throwing away valuable data. In cases where multiple SAGE tags or 2D PAGE spots mapped to a single gene, we summed the tags/spots expressions to arrive at a total expression for the gene. In cases where multiple microarray probes mapped to a single gene, we took the median of the probes’ expressions to arrive at a total expression for the gene.

### Gene and pathway annotation

KEGG (Kyoto Encyclopedia of Genes and Genomes) pathways and GO (Gene Ontology) annotations were retrieved using WebGestalt (Zhang et al. 2005). The most highly represented pathways in the table of correlations with multiple genes (supplementary data) were determined by grouping all genes containing a pathway annotation by the given annotation. Signaling pathways represented by five or more gene members were considered highly represented.

## Results

### Cross-dataset comparisons

In determining how well gene expression correlates across different gene expression datasets, it is not valid to directly compare expression values since different protocols and different normalization methods will result in wide variations in expression values even if the same microarray and biological conditions are used. Where different platforms are used, different pairs of datasets will also have different genes in common. Hence, we chose to use the “correlation of correlations”, or r_c_ (Lee et al. 2003) to assess the degree to which pairwise gene expression correlations compare across each pair of datasets. SAGE expression measurements likely follow a Poisson distribution (Cai et al. 2004), though the often-used Pearson correlation assumes a normal distribution. Thus, we instead use a Spearman rank correlation version of the r_c_, which doesn’t assume any particular distribution, but rather the relative ranks of the expression values (for example if expression values for a set a genes were 5.74, 2.18, 3.65 and 9.13, then their ranks relative to one another would be 3, 1, 2 and 4). The r_c_ between each pair of datasets, computed using the R statistical software (http://www.r-project.org) (Ihaka and Gentleman, 1996), is given in Table 1. The most highly correlated pair of datasets had a correlation value of 0.33. Significance was computed in R by means of permutation testing, which yielded p-values > 0.001 for each pair of datasets except when one of them was the 2D PAGE data set, in which case the p-values ranged from ~0.016 to ~0.574. The relatively low degree of agreement between datasets is not especially surprising in light of published comparisons of mRNA gene expression data from multiple studies involving overlapping or even the same sets of genes (Haverty et al. 2004; Kuo et al. 2002; Tan et al. 2003). These results suggest that inference of gene networks from individual gene expression datasets has to be approached with caution.

### Seed network construction

**Figure 1:**
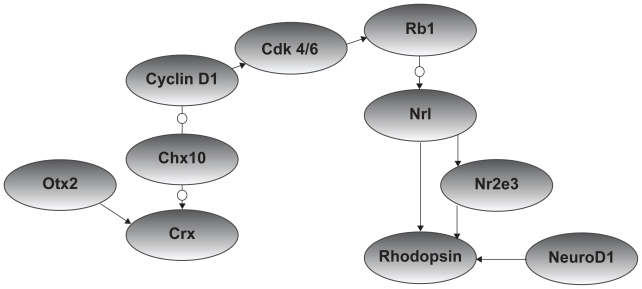
Representation of an intrinsic seed network controlling rod photoreceptor development. The network was constructed based on published experimental evidence and is made up of ten genes. Direct relationships between seed genes are indicated by arrows and indirect relationships are shown as arrows interrupted by circles.

Given the low degree of agreement among the different gene expression datasets, it is natural to question how feasible it is to infer gene networks from gene expression data. In order to address this question, we used an experimentally verified network against which a network inferred from expression data could be validated. We relied on results of experimental studies of retinal development to identify a set of 10 genes that have been implicated in rod photoreceptor development to include in a ‘seed network’ to serve as a basis for validation (Fig. 1). The edges between genes in the network represent several types of links including non-directional interactions inferred from knockout studies (Green et al. 2003; Rutherford et al. 2004) indirect effects on expression inferred from knockout studies (Zhang et al. 2004), phosphorylation events inferred from mutation and transfection experiments (Weinberg, 1995), and direct transcriptional control of one gene by another (Ahmad et al. 1998; Chen et al. 1997; Cheng et al. 2004; Furukawa et al. 2002; Furukawa et al. 1997; Mears et al. 2001; Nishida et al. 2003; Pennesi et al. 2003).

### Reconstruction of seed network from expression data

Having constructed a seed network to serve as a basis for testing the feasibility of inferring gene networks from gene expression data, we proceeded to explore whether the links between the ten seed network genes (Fig. 1) can in fact be reconstructed using one or more gene expression datasets (recall that the links between seed network genes reflect interactions between genes that are supported by published experimental studies).

We examined the pairwise correlations in expression between genes included in the seed network in each of the five mRNA expression datasets. The 2D gel electrophoresis (2DGE) dataset was omitted since none of the seed network genes were identified in it. In this analysis, a link between a pair of seed network genes is supported by a dataset if the corresponding genes were positively or negatively correlated in that dataset, with the absolute value of correlation greater than or equal to 0.65. Our choice of the threshold of 0.65 for correlation was influenced by similar choices in previous studies (Griffith et al. 2005; Gunsalus et al. 2005; Lee et al. 2004) that have revealed biologically relevant links between coexpressed genes. Interestingly, no single dataset supported all nine links in the seed network. Three of the datasets supported six links, one dataset supported four links and one supported three (Table 2).

**Figure 2:**
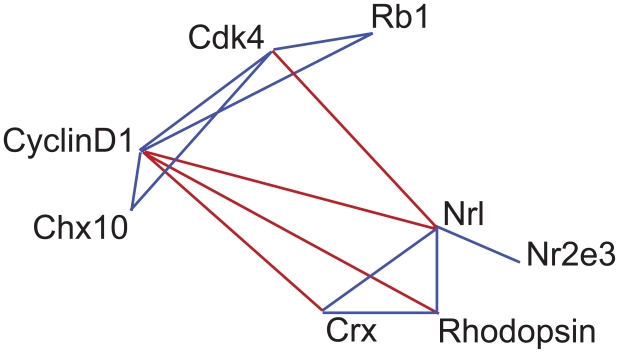
A rod network reconstructed based on correlations among seed genes in the expression datasets. Links were drawn to connect any two seed genes with a correlation of |0.65| or greater in two or more of the five datasets. Blue lines represent positive correlations and red lines represent negative correlations.

We then proceeded to examine whether multiple datasets could be combined to reliably reconstruct the seed network from gene expression data. The resulting network (Fig. 2) shows a link between a pair of seed network genes whenever the pairwise correlation between the expression levels of the corresponding genes is greater than or equal to +0.65 or less than or equal to −0.65 in *at least* 2 of the five datasets. Links depicting positive correlation are shown in blue and those depicting negative correlation are shown in red. Six of the nine ‘positive’ links in this reconstructed network (Fig. 2) are also present as links in the original seed network (Table 2). In addition to the ‘positive’ links there are four ‘negative’ links based on the observed negative correlations between the seed network genes in the reconstructed network. Interestingly, the ‘negative’ links partition the network into two sets of genes, one consisting of genes expressed by proliferating retinal progenitors (Chen and Cepko, 2000; Sicinski et al. 1995; Zhang et al. 2004) and the other consisting of genes expressed by cells in the process of differentiating into rod photoreceptors (Cheng et al. 2004; Furukawa et al. 2002; Mears et al. 2001; Morrow et al. 1998).

The success of this approach in recovering a majority of the links in the seed network, in spite of the relatively low degree of overall agreement among the different datasets (with the largest observed correlation of correlations between any pair of datasets being only 0.33), demonstrates the usefulness of combining multiple gene expression datasets for inferring gene networks from gene expression data and increasing the robustness of the resulting conclusions.

### Prioritizing experimental targets using seed network and expression data

Based on the success of our attempt to (at least partially) recover the links between genes in the seed network, we proceeded to use the seed network to identify additional genes that are likely to be involved in rod differentiation. To do this we queried the gene expression datasets using a procedure similar to the one we used to reconstruct the seed network. For each of our seed genes, we generated a list of all genes whose expression levels were positively or negatively correlated with the network gene in at least two of the five datasets, with the absolute value of the correlation in each case being at least 0.65. We then sorted each list by the number of datasets in which a candidate gene in the list met the correlation threshold of a 0.65 (with a seed network gene) as well as by the mean value of these correlations across those datasets, thus producing a list of prioritized candidate genes correlated with each seed network gene (data not shown).

To further prioritize the candidate genes, we generated a list of genes whose expression levels were positively or negatively correlated with at least two genes of interest (i.e. seed network genes *Nrl*, *Nr2e3*, *Crx, Rb1, Chx10, Rho* and *Neurod1*), and met the correlation threshold of positive (or negative) 0.65 in at least two datasets. Using this approach we identified 986 genes whose expression levels are positively correlated with more than 2 genes of interest with a correlation coefficient of at least 0.65 (Supp. Table 1). We then retrieved Gene Ontology and KEGG pathway annotations for the genes in this list. Based on this information we found the MAPK signaling, oxidative phosphorylation, purine metabolism, glycolysis, gluconeogenesis, tight junction neuroactive ligand-receptor interaction, calcium signaling, and insulin signaling pathway annotations to be prominently represented in this list (Supp. Table 3a and b). Similarly, we identified 531 genes whose expression levels are negatively correlated with more than 2 genes of interest. Based on retrieval of Gene Ontology and KEGG pathway annotations for the genes in this list we found the ribosome, MAPK signaling, cell cycle, axon guidance, regulation of actin cytoskeleton, pyrimidine metabolism, focal adhesion and purine metabolism annotations were prominently represented (Supp. Tables 3c and d).

### Genes with known links to photoreceptors

Several of the genes whose expression levels were found to be highly positively correlated with multiple genes in the rod seed network (based on analysis of more than one data set) are known to be important for rod photoreceptor function, e.g. *phosphodiesterase 6G, cGMP-specific rod gamma, recoverin, rod outer segment membrane protein 1, and phosducin* (Supp. Table 2). The fact that our list of candidate genes includes genes that have strong experimental evidence of involvement in rod photoreceptor functions suggests that the other candidate genes that we have identified through our approach of using a seed network to query multiple expression datasets are worthy of careful consideration in the context of rod development.

### Expanding the seed network into a hypothesized rod gene network

**Figure 3:**
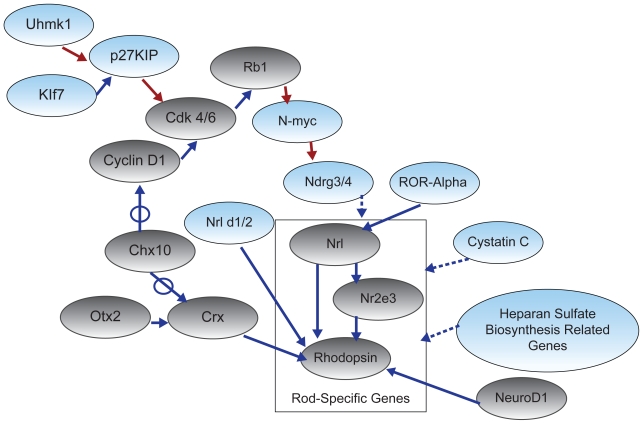
Expansion of the seed network to include candidate genes. Genes highly correlated with multiple seed network members were considered for inclusion into the original seed network. Based on published experimental evidence, seven candidate genes or gene families (represented by blue ovals) were identified and proposed links were added to the seed network genes (represented by gray ovals). Red arrows indicate a negative relationships between genes, blue arrows a positive relationships. The dashed arrows indicate hypothesized links not yet verified by direct experimental evidence. The box surrounding Nrl, Nr2e3, and rhodopsin indicates seed network genes which are specific to rod photoreceptors. Candidate genes (blue), which have a link to this box are proposed to interact (likely indirectly) with several rod genes.

Based on the lists generated by this analysis we have identified seven genes or groups of genes that are candidates for immediate inclusion into a hypothesized ‘rod gene network’, that extends the seed network. These include *Uhmk1, Kruppel-like transcription factor-7, Ext1* and other genes involved in heparan sulfate biosynthesis, *cystatin C, N-myc downstream regulated genes 3 and 4, Nr1d2, and ROR-alpha* (Fig. 3). One additional gene, *p27Kip*, was added to the hypothesized rod gene network based on its interaction with two candidate genes. We also included p27Kip in the hypothesized rod gene network because it inhibits the seed network gene *cdk* and has been shown to regulate retinal progenitor cell cycle withdrawal (Dyer and Cepko, 2001).

U2AF homology motif (UHM) kinase 1, (Uhmk1; also called Kis or Kinase interacting with stathmin), is a serine/threonine kinase that contains an RNA binding motif (Maucuer et al. 1995; Maucuer et al. 1997). *Uhmk1* is positively correlated with *Nrl, Nr2e3, rhodopsin,* and *Crx* and is negatively correlated with *NeuroD1*. Uhmk1 has been found to bind to and negatively regulate the cell cycle inhibitor p27Kip (Boehm et al. 2002), which is involved in regulation of retinal progenitor cell fate. This, together with the observed correlation in *Uhmk1*’s expression with the expression of two well characterized transcription factors that direct photoreceptor cell fate (Crx and Nrl) is highly suggestive of its involvement in rod progenitor cell cycle exit.

Several of the Kruppel-like transcription factors are highly correlated with multiple genes in the rod seed network. The Kruppel-like factors function as repressors or activators of transcription and are good candidates for regulation of genes involved in rod development as they are involved in cell proliferation and differentiation in many tissues including the retina (Otteson et al. 2004). *Kruppel-like transcription factor 7* (*Klf7*) is highly negatively correlated with *Crx* and *Nrl* in multiple datasets. Klf7 is expressed in differentiating cells in the embryonic retina and other parts of the central nervous system (Laub et al. 2001; Laub et al. 2005). *Klf7* knockout mice show downregulation of the cdk inhibitor *p27Kip* and there is evidence that it directly activates the *p27Kip* promoter. Klf7 may therefore play a key role in regulating the cell cycle of retinal progenitors.

Several genes involved with heparan sulfate biosynthesis are correlated with the expression of genes in the seed network. *Exostoses* (*multiple*) *1* or *Ext1* is positively correlated with *Nrl, rhodopsin, Nr2e3* and *Crx. Ext1* is a glycosyltransferase involved in the synthesis of heparan sulfate and is known to be highly expressed in developing mouse brain (Inatani and Yamaguchi, 2003). Other genes involved in heparan sulfate biosynthesis are also highly correlated with multiple genes in our seed network. These include *heparan sulfate* (*glucosamine*) *3-O-sulfotransferase 3B1* which is positively correlated with *Nrl, rhodopsin* and *Nr2e3, beta-1,3-glucuronyltransferase 1* (*glucuronosyltransferase P*) which is positively correlated with *Nrl* and *rhodopsin,* and *carbohydrate* (*chondroitin*) *synthase 1* which is also positively correlated with *Nrl* and *rhodopsin*. A role for heparan sulfate in retinal development has been suggested by studies of its expression and heparan sulfate has been shown to have an effect on several pathways important in development such as the hedgehog and fibroblast growth factor pathways (Cool and Nurcombe, 2006; Rubin et al. 2002).

*Cystatin C* is positively correlated with *Nrl, Nr2e3, Crx,* and *rhodopsin*. Cystatin C is a cysteine protease inhibitor found in many tissues including the retina. Cystatin C RNA and protein expression have been detected in the embryonic and postnatal rodent retina with peak levels of the protein expressed around the time of photoreceptor maturation (Barka and Van Der Noen, 1994; Wasselius et al. 2001). Recently, Kato et al. (Kato et al. 2006) isolated cystatin C from conditioned media of primary neurospheres and demonstrated that addition of cystatin C to embryonic stem cells facilitated the differentiation into cells expressing neural genes. The fact that *cystatin C* is expressed in the developing retina, is implicated in promoting neuronal cell fate determination, and is correlated with multiple seed network genes makes it a likely candidate for involvement in photoreceptor development.

*N-myc downstream regulated gene 3* (*Ndrg3*) is highly positively correlated with *Crx, Nrl,* and *rhodopsin*. Another N-myc downstream regulated gene, *Ndrg4 is* highly correlated with *Nrl* in two datasets. *Ndrg3* and *Ndrg4* are inhibited by N-myc, one of the members of the myc family of protooncogenes. *N-myc* has been shown to be important in central nervous system development and is thought to play a role in CNS cell proliferation and differentiation (Stanton et al. 1992). *N-myc* is highly negatively correlated with *Nrl* and *rhodopsin. N-myc* is expressed in the developing retina but not in mature retinal neurons (Hirning et al. 1991). N-myc is inhibited by retinoblastoma (*Rb1*) and expression of *Ndrg3* and *Ndrg4* are reduced in the Rb knockout retina (data accessible at NCBI GEO database, accession number GSE1129; http://www.ncbi.nlm.nih.gov/geo/query/acc.cgi?acc=GSE1129). Therefore Rb1 may be important for inhibition of N-myc during cell fate determination in the retina which in turn increases expression of *Ndrg3* and *Ndrg4*. Ndrg3 and Ndrg4 may promote rod differentiation through enhancement of AP-1 activity as Ndrg4 has been shown to regulate activity of the protein complex (Ohki et al. 2002). AP-1 binding sites are found in the Nrl promoter region and the promoters of other rod specific genes (Farjo et al. 1993).

The orphan nuclear receptor *Nr1d2* is highly correlated with *Crx, Nrl, Nr2e3* and *rhodopsin*. This gene is a member of the Reverb nuclear receptor subgroup along with Reverb alpha (Nr1d1), which can function as transcriptional silencers and can repress transcriptional activation by retinoid-related orphan receptor alpha (*Nr1f1*) and thyroid hormone receptor (Forman et al. 1994). There is evidence that Nr1d1 interacts with Nr2e3 and Nrl to activate transcription of rhodopsin in the retina (Cheng et al. 2004). Both Reverb proteins bind to the same core promoter sequence suggesting that Nr1d2 may also be involved in activating transcription of rhodopsin and other rod photoreceptor genes.

Another orphan nuclear receptor highly correlated with the rod seed genes *Nrl* and *Crx* was *retinoid-related orphan receptor alpha* (*ROR-alpha*). ROR-alpha is a member of the steroid/thyroid hormone receptor superfamily. Interestingly it has recently been shown that *Nrl* contains a putative ROR-alpha response element and other retinoic acid receptor binding sites in its promoter region and that deletion of these elements decreases retinoic acid induced luciferase activity in Nrl promoter-luciferase constructs (Khanna et al. 2006). Discovering the ligands for ROR-alpha and the Reverb nuclear receptors could reveal factors important for controlling Nrl expression in developing photoreceptors. Examination of the data extracted from the mouse retina SAGE library (http://itstgp01.med.harvard.edu/retina) suggests that ROR-alpha is more highly expressed in the outer nuclear layer of the retina than retinoic acid receptor alpha (RAR-alpha) and its temporal RNA expression more closely correlates with that of *Nrl*.

### Summary of candidate genes

The information available in literature on the candidate genes summarized above makes them likely candidates for linking with specific genes in the rod seed network (Fig. 3). Both *Uhmk1* and *Klf7* may be involved in rod genesis through regulation of cell cycle progression by negative or positive regulation of *p27Kip*. The orphan nuclear protein *ROR-alpha* is linked directly to *Nrl* based on a putative binding site present in the *Nrl* promoter region. *Nr1d2* is linked to rhodopsin based on its similarities to *Nr1d1*, a protein that is known to bind to the rhodopsin promoter region. *Ndrg* 3 and 4, genes involved in heparan sulfate biosynthesis, and cystatin C correlated with several rod genes, and are shown to have links with all rod specific genes.

Recently, efforts to identify members of the photoreceptor transcriptional network used mouse knockouts of *Nrl, Nr2e3* and *Crx* to identify genes that may be regulated by, and therefore primarily downstream of these three key transcription factors (Hsiau et al. 2007). Of the 628 genes dysregulated genes identified by this study, 174 are present in our list of 1789 genes either positively or negatively correlated with multiple seed network members. Our results are complimentary to this study, as our approach is likely to identify candidates upstream of *Crx, Nrl* and *Nr2e3* as well.

## Discussion

Several large-scale gene expression studies of the murine retina have been conducted in an attempt to identify genes important for retinal development (Akimoto et al. 2006; Blackshaw et al. 2004; Dorrell et al. 2004; Liu et al. 2006; Mu et al. 2001; Zhang et al. 2006). The data from these studies provide useful information about the changes in gene expression during retinal development. However, these studies offer at best only a starting point for functional studies focused on a smaller subset of genes. The relatively low degree of correspondence in terms of pairwise correlations in gene expression across datasets from different studies further complicates the use of multiple datasets to extract a small subset of the genes as good candidates for a role in specific events in retinal development (such as rod photoreceptor genesis).

Against this background, we have explored a novel approach for analysis of multiple gene expression datasets to identify genes that are likely to play important roles in rod photoreceptor development. We have demonstrated a simple approach to leveraging multiple gene expression datasets to increase the robustness of inferred links between genes, by focusing on links supported by multiple gene expression datasets. We then used a similar approach to query multiple gene expression datasets, using a seed network consisting of a small number of genes (known to be important in rod development), to identify genes whose expression levels are highly correlated with those of the seed network genes in multiple datasets.

The simple approach to combine information from multiple gene expression datasets, used here, does not assign different weights to the evidence provided by the different datasets. It might be useful to consider more robust approaches to leveraging information from multiple gene expression datasets e.g. using a machine learning algorithm (Baldi and Brunak, 2001) to *learn* the weights to be used to combine the evidence provided by the different datasets in support of links between seed network genes and other genes in the datasets. For example, the weights could be optimized using machine learning so as to maximize the accuracy of reconstruction of the seed network from the available data. The resulting weights could then be used in expanding the seed network by adding new links based on evidence from multiple datasets.

The hypothesized rod network described here summarizes our first results obtained using the approach developed in this paper for querying multiple gene expression datasets using a seed network. Our analysis has focused on narrowing down the list of 986 genes that are positively correlated with at least 2 seed network genes. We have not yet analyzed the list of 531 genes that are negatively correlated with at least 2 seed network genes. Of particular interest are genes that are positively correlated with some seed network genes and negatively correlated with other seed network genes. We have relied mostly on the analysis of Gene Ontology and KEGG pathway annotations of genes that are correlated with at least 2 seed network genes in the broader context of the current literature on retinal development. Several additional sources of information can be brought to bear on the task of further refining the hypothesized rod gene network, e.g. protein-protein interaction data, phosphorylation data, among others. Work in progress is aimed at exploring some of these directions.

## Related Work

Several previous studies have examined ways of extending a known seed network (Bader, 2003; Cabusora et al. 2005; Can et al. 2005; Dougherty et al. 2000; Hashimoto et al. 2004; Shmulevich et al. 2002). Most of these focus on filtering or selecting candidate links based on some criteria (Bader, 2003; Cabusora et al. 2005; Dougherty et al. 2000; Hashimoto et al. 2004; Shmulevich et al. 2002) or producing a single ranking of all genes in terms of the degree to which they are “related” to the entire seed network (Can et al. 2005). In contrast, we focus on producing a ranking for each seed gene as well as a ranking of those genes that are correlated with multiple seed genes. The latter is especially useful in showing, at a glance, the specific genes in the seed network that are likely to be involved in interactions with a candidate gene. The resulting prioritized list can then be further examined by human experts in the broader context of related literature and biological knowledge.

## Summary

By using a seed network to query multiple retinal gene expression datasets we were able to identify candidate genes for further study related to rod photoreceptor development. We used the seed network to prioritize genes in the datasets based on their correlation with multiple seed gene members. Based on further analysis of the prioritized lists in the context of evidence obtained from the literature in support of the new links, we were able to identify a small subset of genes from the prioritized lists for addition to the seed network. These new links in the resulting rod gene network offer a rich source of hypotheses that can help focus the experiments at the bench. We believe that this approach offers a powerful means of leveraging computational analysis of high-throughput gene expression data, together with the interpretation of the results by biologists in the context of existing biological knowledge, to rapidly identify and prioritize experimental targets.

## Supplementary Materials

Supplementary Table 1 Genes the correlate with multiple seed genes. Genes that correlate with multiple seed genes (correlation value of 0.65 or greater in at least two datasets) are listed. A correlation of 0 in this table indicates that the gene was not present in a particular dataset.

Supplementary Table 2 Photoreceptor genes that correlate with multiple seed genes. This contains the subset of genes from Supplementary Table 1 that are expressed in photoreceptors. For each gene that is listed, the correlated seed gene is indicated as well as the mean correlation across datasets in which the correlation reached threshold.

Supplementary Table 3: 3a: KEGG annotations retrieved using the list of genes positively correlated with multiple rod seed network genes. This table lists the number of times an annotation was retrieved.

3b: This list contains genes positively correlated with multiple seed network genes that also have an annotation linking them to a pathway. Genes are listed by their Unigene symbol and are grouped according to the signaling pathways with which they are associated.

3c: KEGG annotations retrieved using the list of genes negatively correlated with multiple rod seed network genes. This table lists the number of times an annotation was retrieved.

3d: This list contains genes negatively correlated with multiple seed network genes that also have an annotation linking them to a pathway. Genes are listed by their Unigene symbol and are grouped according to the signaling pathways with which they are associated.

## Figures and Tables

**Table 1:** Correlations of correlations values between each of the gene expression datasets. In calculating each correlation of correlations, only the subset of genes in common between the two datasets was used. This subset was different for each pair of datasets. SAGE = SAGE data from whole retina (Blackshaw et al. 2004); MOE430.2.0 = Affymetrix microarray data from developing rod progenitors (Akimoto et al. 2006); Mu74Av2_1 = Affymetrix microarray data from whole retina (Dorrell et al. 2004); Mu74Av2_2 = Affymetrix microarray data from whole retina (Liu et al. 2006); cDNA microarray = cDNA microarray data from whole retina (Zhang et al. 2006); 2DGE = 2D-PAGE data from whole retina (Barnhill and Greenlee, personal communication). * p < 0.001, ** p < 0.02, *** p < 0.05.

	SAGE	MOE430.2.0	Mu74Av2_1	Mu74Av2_2	cDNA microarray	2DGE
SAGE		0.1*	0.23*	0.12*	0.09*	0.05
MOE430.2.0	0.1*		0.18*	0.09*	0.04*	0
Mu74Av2_1	0.23*	0.18*		0.33*	0.09*	0.07
Mu74Av2_2	0.12*	0.09*	0.33*		0.02*	0.06
cDNA microarray	0.09*	0.04*	0.09*	0.02*		0.06
2DGE	0.05***	0	0.07**	0.06**	0.06	

* p < 0.001

** p < 0.02

*** p < 0.05

**Table 2:** Datasets supporting each positive edge between all pairs of genes shown to be linked in Figure 2. Datasets supporting a particular link between seed genes (based on correlation) are marked with an X. The last column indicates whether that edge was present in the network based on the literature (Fig. 1).

	SAGE	MOE430.2.0	Mu74Av2_1	Mu74Av2_2	cDNA microarray	Original Seed Network
CyclinD1-Cdk4		X	X	X	X	Yes
CyclinD1-Chx10				X	X	Yes
CyclinD1-Rb1		X		X		No
Cdk4-Rb1		X	X		X	Yes
Cdk4-Chx10				X	X	No
Crx-Nrl	X		X			No
Nrl-Nr2e3	X	X			X	Yes
Nrl-Rhodopsin	X	X		X	X	Yes
Crx-Rhodopsin	X	X				Yes
