# Supplementary material for: Using a Seed-Network to Query Multiple Large-Scale Gene Expression Datasets from the Developing Retina in Order to Identify and Prioritize Experimental Targets
Source: Bioinform Biol Insights. 2008 Feb 1;2:401–12. doi: 10.4137/bbi.s417 (PMC2735966; doi:10.4137/bbi.s417)
Supplement: Supplementary Table 2 — Photoreceptor genes that correlate with multiple seed genes. This contains the subset of genes from Supplementary Table 1 that are expressed in photoreceptors. For each gene that is listed, the correlated seed gene is indicated as well as the mean correlation across datasets in which the correlation reached threshold. [file Supp_table_2.pdf]

Seed gene	Mean correlation	Gene symbol	Gene name
Nrl	0.923	Arr3	arrestin 3, retinal
Rho	0.994	Arr3	arrestin 3, retinal
Crx	0.727	Aipl1	aryl hydrocarbon receptor-interacting protein-like 1
Rho	0.89	Aipl1	aryl hydrocarbon receptor-interacting protein-like 1
Nr2e3	0.687	Aipl1	aryl hydrocarbon receptor-interacting protein-like 1
Nrl	0.838	Aipl1	aryl hydrocarbon receptor-interacting protein-like 1
Rho	0.889	Abca4	ATP-binding cassette, sub-family A (ABC1), member 4
Crx	0.679	Abca4	ATP-binding cassette, sub-family A (ABC1), member 4
Nrl	0.921	Abca4	ATP-binding cassette, sub-family A (ABC1), member 4
Nrl	0.836	Bbs2	Bardet-Biedl syndrome 2 homolog (human)
Rho	0.823	Bbs2	Bardet-Biedl syndrome 2 homolog (human)
Crx	0.794	Bbs2	Bardet-Biedl syndrome 2 homolog (human)
Nrl	0.937	Cacna1f	calcium channel, voltage-dependent, alpha 1F subunit
Crx	0.811	Cacna1f	calcium channel, voltage-dependent, alpha 1F subunit
Nr2e3	0.804	Cacna1f	calcium channel, voltage-dependent, alpha 1F subunit
Rho	0.931	Cacna1f	calcium channel, voltage-dependent, alpha 1F subunit
Rho	0.962	Crb1	crumbs homolog 1 (Drosophila)
Nrl	0.923	Crb1	crumbs homolog 1 (Drosophila)
Rho	0.942	Cnga1	cyclic nucleotide gated channel alpha 1
Crx	0.78	Cnga1	cyclic nucleotide gated channel alpha 1
Nrl	0.892	Cnga1	cyclic nucleotide gated channel alpha 1
Rho	0.75	Gabrr2	gamma-aminobutyric acid (GABA-C) receptor, subunit rho 2
Nrl	0.695	Gabrr2	gamma-aminobutyric acid (GABA-C) receptor, subunit rho 2
Crx	0.81	Gja9	gap junction membrane channel protein alpha 9
Nrl	0.89	Gja9	gap junction membrane channel protein alpha 9
Nrl	0.799	Gnat1	guanine nucleotide binding protein, alpha transducing 1
Rho	0.837	Gnat1	guanine nucleotide binding protein, alpha transducing 1
Crx	0.735	Gnat1	guanine nucleotide binding protein, alpha transducing 1
Rho	0.926	Gnat2	guanine nucleotide binding protein, alpha transducing 2
Nrl	0.913	Gnat2	guanine nucleotide binding protein, alpha transducing 2
Rho	0.789	Guca1a	guanylate cyclase activator 1a (retina)
Nrl	0.815	Guca1a	guanylate cyclase activator 1a (retina)
Nrl	0.691	Guca1b	guanylate cyclase activator 1B
Rho	0.817	Guca1b	guanylate cyclase activator 1B
Crx	0.931	Pdc	phosducin

Rho	0.841	Pdc	phosducin
Nrl	0.882	Pdc	phosducin
Nr2e3	0.823	Pdc	phosducin
Rho	0.875	Pde6a	phosphodiesterase 6A, cGMP-specific, rod, alpha
Crx	0.709	Pde6a	phosphodiesterase 6A, cGMP-specific, rod, alpha
Nrl	0.761	Pde6a	phosphodiesterase 6A, cGMP-specific, rod, alpha
Nrl	0.914	Pde6b	phosphodiesterase 6B, cGMP, rod receptor, beta polypeptide
Nr2e3	0.849	Pde6b	phosphodiesterase 6B, cGMP, rod receptor, beta polypeptide
Rho	0.925	Pde6b	phosphodiesterase 6B, cGMP, rod receptor, beta polypeptide
Nrl	0.896	Pde6g	phosphodiesterase 6G, cGMP-specific, rod, gamma
Crx	0.784	Pde6g	phosphodiesterase 6G, cGMP-specific, rod, gamma
Rho	0.943	Pde6g	phosphodiesterase 6G, cGMP-specific, rod, gamma
Nr2e3	0.804	Pde6g	phosphodiesterase 6G, cGMP-specific, rod, gamma
Crx	0.883	Ppef2	protein phosphatase, EF hand calcium-binding domain 2
Rho	0.728	Ppef2	protein phosphatase, EF hand calcium-binding domain 2
Nrl	0.862	Ppef2	protein phosphatase, EF hand calcium-binding domain 2
Nr2e3	0.797	Pcdh21	protocadherin 21
Rho	0.803	Pcdh21	protocadherin 21
Nrl	0.838	Pcdh21	protocadherin 21
Nr2e3	0.799	Rcvrn	recoverin
Nrl	0.906	Rcvrn	recoverin
Rho	0.898	Rcvrn	recoverin
Crx	0.775	Rcvrn	recoverin
Rho	0.894	Rgs9bp	regulator of G-protein signalling 9 binding protein
Nrl	0.773	Rgs9bp	regulator of G-protein signalling 9 binding protein
Rho	0.878	Rds	retinal degeneration, slow (retinitis pigmentosa 7)
Nrl	0.917	Rds	retinal degeneration, slow (retinitis pigmentosa 7)
Nrl	0.901	Sag	retinal S-antigen
Nr2e3	0.862	Sag	retinal S-antigen
Rho	0.897	Sag	retinal S-antigen
Rho	0.84	Rlbp1	retinaldehyde binding protein 1
Nrl	0.866	Rlbp1	retinaldehyde binding protein 1
Nr2e3	0.786	Rlbp1	retinaldehyde binding protein 1
Rho	0.832	Rp1h	retinitis pigmentosa 1 homolog (human)
Nr2e3	0.892	Rp1h	retinitis pigmentosa 1 homolog (human)
Nrl	0.975	Rp1h	retinitis pigmentosa 1 homolog (human)
Nrl	0.927	Rp1hl1	retinitis pigmentosa 1 homolog (human)-like 1
Rho	0.972	Rp1hl1	retinitis pigmentosa 1 homolog (human)-like 1

Crx	0.786	Rpgrip1	retinitis pigmentosa GTPase regulator interacting protein 1
Nr2e3	0.834	Rpgrip1	retinitis pigmentosa GTPase regulator interacting protein 1
Rho	0.977	Rpgrip1	retinitis pigmentosa GTPase regulator interacting protein 1
Nrl	0.891	Rpgrip1	retinitis pigmentosa GTPase regulator interacting protein 1
Crx	0.786	Rbp3	retinol binding protein 3, interstitial
Rho	0.871	Rbp3	retinol binding protein 3, interstitial
Nrl	0.828	Rbp3	retinol binding protein 3, interstitial
Crx	0.746	Rdh12	retinol dehydrogenase 12
Nr2e3	0.799	Rdh12	retinol dehydrogenase 12
Rho	0.951	Rdh12	retinol dehydrogenase 12
Nrl	0.901	Rdh12	retinol dehydrogenase 12
Rho	0.95	Rom1	rod outer segment membrane protein 1
Crx	0.821	Rom1	rod outer segment membrane protein 1
Nrl	0.931	Rom1	rod outer segment membrane protein 1
Nr2e3	0.839	Rom1	rod outer segment membrane protein 1
Rho	0.861	Slc24a1	solute carrier family 24 (sodium/potassium/calcium exchanger), member 1
Nrl	0.814	Slc24a1	solute carrier family 24 (sodium/potassium/calcium exchanger), member 1
Nrl	0.859	Tub	tubby candidate gene
Rho	0.844	Tub	tubby candidate gene
Crx	0.75	Tub	tubby candidate gene
Crx	0.901	Tulp1	tubby like protein 1
Nrl	0.903	Tulp1	tubby like protein 1
Rho	0.901	Tulp1	tubby like protein 1
Rho	0.899	Unc119	unc-119 homolog (C. elegans)
Nrl	0.823	Unc119	unc-119 homolog (C. elegans)
Crx	0.794	Unc119	unc-119 homolog (C. elegans)
Nr2e3	0.7	Unc119	unc-119 homolog (C. elegans)
